# The safety and efficacy of nonvitamin K antagonist oral anticoagulants in morbidly obese patients with atrial fibrillation: a meta-analysis

**DOI:** 10.1186/s12872-024-03731-3

**Published:** 2024-01-26

**Authors:** Sohil Elfar, Somaya Abdulbaset Mahmoud, Samar Hamdi, Aya Ahmed Emad, Mohamed Abd-ElGawad, Nouran A. Taha

**Affiliations:** 1 Port Said University, Port Said, Egypt; 2 Faculty of Pharmacy, October 6th University, Cairo, Egypt; 3 Faculty of Pharmacy, Mansoura University, Mansoura, Egypt; 4 Nutrition support pharmacist, CCHE57357, Cairo, Egypt; 5 Faculty of Medicine, Elfayoum University, Elfayoum, Egypt; 6 MARS-Global, London, UK

**Keywords:** Nonvitamin K antagonist oral anticoagulants, Warfarin, Atrial fibrillation, Morbid obesity

## Abstract

**Background and aim:**

Atrial fibrillation (AF) is the most frequently observed cardiac arrhythmia in clinical settings. Obesity can influence the efficacy of the treatment administered, which requires a larger dose and more time to accomplish therapeutic targets due to altered pathophysiology. Our study aimed to assess the overall efficacy and safety of nonvitamin K antagonist oral anticoagulants (NOACs) versus warfarin in AF patients with morbid obesity (BMI > 40 kg/m2 and/or weight > 120 kg) to prevent complications.

**Methods:**

We conducted a literature search on PubMed, Web of Science, the Cochrane Library, and Scopus till October 2022 for articles addressing the efficacy and safety of NOACs versus warfarin for the treatment of AF in morbidly obese patients. We performed the meta-analysis with RevMan software version 5.4 and Open Meta Analyst. The main outcomes assessed were stroke, major bleeding, and minor bleeding after anticoagulation, as did the history of comorbidities and risk factors in morbidly obese patients. Quality assessment was performed using Cochrane’s ROB-2 tool and the Newcastle–Ottawa scale.

**Results:**

Regarding major bleeding events, pooled data showed that patients taking NOACs had a significantly lower risk than patients taking warfarin (OR = 0.54, 95% CI: [0.41–0.70]; *p* < 0.00001). However, for minor bleeding, there was a nonsignificant effect of NOACs on reducing the risk of bleeding (OR = 0.72, 95% CI = 0.47–1.09; *p* = 0.12), which became highly significant in favor of NOACs after sensitivity analysis (OR = 0.55, 95% CI = 0.49–0.61]; *p* < 0.00001). There was a significant difference in the incidence of stroke between the NOAC group and the warfarin group (OR = 0.69, 95% CI = 0.60–0.80]; *p* < 0.00001). According to the results of the single-arm study analysis, the overall effect of all the outcomes was associated with a high risk of disease development in patients receiving NOACs.

**Conclusion:**

Our meta-analysis showed a favorable effect of NOACs vs warfarin in morbidly obese patients. Some outcomes were not significantly different, which calls for future research to better assess their safety and efficacy in this particular weight group.

**Trial registration:**

The study was registered with PROSPERO under registration number CRD42022362493 on October 2022.

**Supplementary Information:**

The online version contains supplementary material available at 10.1186/s12872-024-03731-3.

## Introduction

Atrial fibrillation (AF) is the most persistent and frequently observed cardiac arrhythmia in clinical settings. It has been associated with a higher risk of death, stroke, and peripheral embolism [1]. The prevalence of AF varies depending on age, from 2% in the general population to 10–12% in people who are 80 years or older [2].

The onset and persistence of atrial fibrillation are both significantly influenced by obesity (AF). Compared to nonobese people, obese people have a nearly 50% increased risk of developing AF [3]. According to estimates, obesity causes nearly one in five cases of AF, with each incremental increase in body mass index (BMI) increasing the incidence of the condition by 4 to 5% [4]. Obesity can additionally influence the efficacy of the treatment administered due to differences in the metabolism and distribution of the drugs, which in turn can cause additional complications.

All international guidelines strongly advise the use of anticoagulants for AF patients at high risk of stroke (CHA2DS2-VASc score ≥ 2) [5, 6]. These recommendations advocate for the use of nonvitamin K antagonist oral anticoagulants (NOACs) rather than warfarin due to the significant connection with serious bleeding, numerous food and drug interactions, and the requirement for ongoing monitoring [7, 8]. Compared to adults of normal weight, obese adults may require a greater dose and more time to accomplish therapeutic targets due to altered pathophysiology that can affect the pharmacology of anticoagulants, including warfarin [9]. Due to anticoagulant underdosing, this may contribute to adverse events such as stroke and hospitalization.

The International Society of Thrombosis and Hemostasis advises against using NOACs among individuals with a BMI > 40 or > 120 kg, although they recommend the standard dosing of NOACs in patients with obesity and with a BMI below 40 kg or weight below 120 kg due to the paucity of clinical data for these patients [10]. The utility of NOACs in patients with morbid obesity has not been thoroughly studied or established. Therefore, it is critical to compare the risk of stroke and major bleeding (MB) among other serious adverse effects in patients taking nonvitamin K antagonist oral anticoagulants who suffer from AF and are morbidly obese.

Despite the well-known negative effects of obesity on cardiovascular health, a paradoxical phenomenon known as the obesity paradox has been theorized about in several systematic reviews and meta-analyses. According to this phenomenon, studies with longer-term follow-up periods showed that participants who were overweight or mildly obese (BMI 35 kg/m2) and in the NOAC group had lower all-cause mortality [11, 12]. Despite these findings, several studies have criticized this assumption due to the possibility of unfounded associations with rhythm control strategies, unreported confounders, or selection bias in observational or cohort studies [13–15].

NOACs have drawn attention in several systematic reviews investigating their use in individuals with obesity [16–32]. The results of published studies on the efficacy and safety of NOACs in obese patients are variable. Barakat et al. reported that, compared with warfarin, NOACs were associated with a 25% reduction in the risk of ischaemic stroke, an approximately 60% reduction in the risk of bleeding events, and an approximately 50% reduction in the risk of haemorrhagic stroke [27]. However, a post-hoc analysis of the Apixaban for Reduction in Stroke and Other Thromboembolic Events in Atrial Fibrillation (ARISTOTLE) trial claimed that apixaban and warfarin both carry comparable relative risks of severe bleeding, despite apixaban having a reduced absolute risk [22].

Since the results of multiple studies have divergent recommendations, we aimed to conduct a systematic review and meta-analysis to investigate the overall effect of NOACs versus warfarin in AF patients with morbid obesity (BMI > 40 kg/m2 and/or weight > 120 kg).

## Methods

This systematic review and meta-analysis were conducted according to the Preferred Reporting Items for Systematic reviews and Meta-Analyses (PRISMA) statement guidelines, and all steps were performed with strict adherence to the Cochrane Handbook of Systematic Reviews and Meta-analysis [33, 34]. The study was registered with PROSPERO under registration number CRD42022362493.

### Literature search strategy

We searched the following medical electronic databases: PubMed, Web of Science, the Cochrane Library, and Scopus up to October 2022. The detailed search strategy is presented in Supplementary File 1.

### Inclusion and exclusion criteria

Two independent authors screened the articles in two steps: title/abstract screening and full-text screening. Any conflicts were resolved by consensus or group discussion. We included both experimental (including a post hoc analysis of a randomized controlled trial (RCT)) and observational studies (either prospective or retrospective) with the following criteria: population of patients (≥18 years of age) with morbid obesity (BMI > 40 kg/m2 and/or weight > 120 kg) and the use of NOACs and/or warfarin for AF treatment. The exclusion criteria included not morbidly obese patients, postablation patients, patients with venous thromboembolism, patients not on NOACs, and patients without atrial fibrillation. Additionally, studies with incomplete data, case reports, review articles, editorials, guidelines, duplicates, or not in the English language were excluded.

### Data extraction

One independent author extracted the data from the included studies using a Google sheet. The data extraction sheets included the following information: study design; country, intervention and comparator; follow-up period; patient characteristics, including history of comorbidities; and main outcomes. Additionally, we extracted the CHA2D2-VASc score from relevant studies. The outcomes assessed were stroke, embolization, major or minor bleeding, and death.

### Methodological quality assessment

Since our systematic review and meta-analysis included different study designs, multiple quality assessment tools were used. For the RCTs, we used version 2 of the Cochrane risk-of-bias tool for randomized trials (RoB-2) to assess bias in the randomization process, deviations from the intended interventions, missing outcome data, measurement of the outcome, and selection of the reported result [35].

For retrospective cohort studies, the Newcastle–Ottawa scale (NOS) was used for assessment [36]. The NOS is based on a star scoring system in which a maximum of nine (for cohort and cross-sectional studies) or ten scores (for case–control studies) can be awarded to each study. The quality assessment was independently checked by one author and reviewed by all the authors to resolve any disagreements.

### Strategy for data synthesis and statistical analysis

The data from the included studies were pooled and analysed with RevMan V.5.4 software. For the analysis of single-arm studies, Open Meta Analyst was used [37].

We used forest plots for improved data visualization. We used the chi-square test to assess heterogeneity. The impact of each study on the pooled analysis was examined using sensitivity analysis. Pooled treatment effects for binary endpoints were compared using odds ratios (ORs) with 95% confidence intervals (CIs). For continuous variables, mean differences (MDs) with 95% CIs were calculated. The overall *p* value was considered to be significant when p was < 0.05. Heterogeneity was examined with the Cochran Q test and was considered to be statistically significant when the two-tailed p value was < 0.05. Since heterogeneity was high, a random-effects model was used, followed by sensitivity analysis using the “leave-one-out” test. A funnel plot was generated and inspected visually to determine the possibility of publication bias in the hypertension subgroup.

## Results

### Study selection

**Fig. 1:**
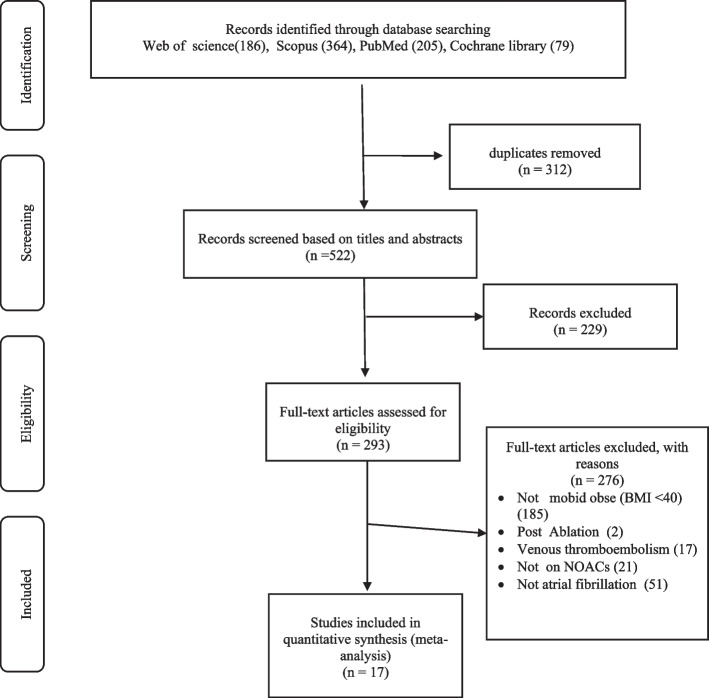
The preferred reference items for systematic reviews and meta-analysis (PRISMA) flow chart depicting the screening process for the included studies

We conducted a literature search of PubMed, Web of Science, the Cochrane Library, and Scopus from inception until the end of 2022 for relevant articles addressing the efficacy of nonvitamin K antagonist oral anticoagulants (NOACs) in comparison to warfarin for the treatment of atrial fibrillation (AF) in morbidly obese patients (BMI > 40 kg/m2 and/or weight > 120 kg). A total of 834 records were recovered, 312 of which were removed as duplicates. Finally, seventeen studies [16–32] were eligible for inclusion in our systematic review and meta-analysis. A detailed PRISMA diagram illustrating the study selection steps is presented in Fig. 1.

### Baseline characteristics of the included studies

A total of 17 studies [16–32] were eligible for inclusion in our systematic review and meta-analysis. A total of 142,256 patients were included, and the mean age was 65.7 years. A summary of the included studies and their baseline characteristics can be found in Table 1.

**Table 1:** Summary of included studies and their baseline characteristics. (NR = Not Reported)

Author Name and Study Date	Study Design	Treatment group (number of patients)	Mean Age (SD)	Sex (female)	CHA2D2VASC Score Mean (SD)	Prior Stroke or Embolization	Heart Failure	Hypertension	Diabetes Mellitus
Briasoulis 2021 [26]	Retrospective study	Apixaban (*n* = 4471)	69.9	1%	NR	7%	31.1%	84.9%	22%
		Dabigatran (*n* = 3246)	65.7	1%	NR	5%	26.2%	84.5%	29.1%
		Rivaroxaban (*n* = 3299)	66.7	1%	NR	4.5%	27.7%	83.2%	25.9%
		Warfarin (*n* = 10,338)	66.5	1.1%	NR	7.3%	35.8%	86.8%	31.8%
Huang 2021 [30]	Retrospective study	Dabigatran (*n* = 3226)	66.4 (59.7,72.0)	1128 (35%)	NR	135 (4.2%)	1137 (35.2%)	1241 (38.5%)	1563 (48.5%)
		Warfarin (*n* = 3622)	66.3 (58.9,72.6)	1333 (36.8%)	NR	138 (3.8%)	1482 (40.9%)	3066 (84.6%)	2011 (55.5%)
Peterson 2019 [19]	Retrospective cohort	Rivaroxaban (*n* = 4543)	61.8 (10.8)	2046 (45.0	NR	NR	1397 (30.8%)	3962 (87.2%)	2168 (47.7%)
		Warfarin (*n* = 4931)	64.4 (10.8)	2326 (47.2)	NR	NR	2218 (45.0%)	4348 (88.2%)	2841 (57.6%)
Navarro-Almenzar 2021 [25]	Retrospective study	Rivaroxaban (*N* = 54)	71 ± 11	82 (60.7%)	NR	21 (15.6%)	29 (21.5%)	124 (91.6%)	51 (37.8%)
		Apixaban (*n* = 42)							
		Dabigatran (*n* = 39)							
		Edoxaban (*n* = 0)							
Barakat 2021 [28]	Retrospective study	Apixaban (*n* = 983)	64.9 ± 9.8	902 (51.4)	NR	205 (9.5)	564 (26.0)	1743 (80.3)	935 (43.1)
		Rivaroxaban (*n* = 861)							
		Dabigatran (*n* = 322)							
		Edoxaban (*n* = 4)							
		Warfarin (*N* = 1754)	66.6 ± 10.2	1189 (54.8)		142 (8.1)	579 (33.0)	1354 (77.2	864 (49.3)
Kushnir 2019 [21]	Retrospective study	Apixaban (*n* = 103)	65·9 (10·7)	58 (56%)	3·5 (1·6)	NR	NR	NR	NR
		Rivaroxaban (*n* = 174)	60·9 (12·6)	95 (55%)	3·1 (1·5)	NR	NR	NR	NR
		Warfarin (*n* = 152)	66·8 (13·6)	90 (59%)	4.1 (1·8)	NR	NR	NR	NR
Boriani 2019 [20]	Randomized, double blind, double-dummy study c	Edoxaban (*n* = 415)	64 ± 8.9269	153	3.3 ± 1.6	NR	NR	NR	NR
		Warfarin (*N* = 364)	64 ± 8.9269	90	4.1 ± 1.8	NR	NR	NR	NR
Bodega 2021 [27]	Retrospective study	Dabigatran (*n* = 7)	59.7 ± 11.6	2	NR	0	5	13	5
		Rivaroxaban (n = 4)							
		Apixaban (*n* = 4)							
		Edoxaban (*n* = 1)							
Hohnloser 2019 [22]	Randomized, double blind, double-dummy study	Apixaban and warfarin (*N* = 982)	61.6498 ± 8.1664	154	NR	96	365	919	449
Kido 2018 [18]	Retrospective study	Dabigatran (*N* = 20)	64.28 ± 10.16	39 (60.84%)	NR	12	NR	NR	NR
		Rivaroxaban (*n* = 25)							
		Apixaban (*n* = 19)							
		Warfarin (*N* = 64)	65.88 ± 12.18	35 (54.69%)	NR	10	NR	NR	NR
Sulaiman 2022 [33]	Retrospective cohort study	Apixaban (*N* = 127)	68.3 ± 10.46	111	NR	15	62	116	94
O’Kane 2022 [32]	Retrospective cohort study	apixaban and rivaroxaban (*N* = 299)	62 ± 11.9188	188	4 ± 1.4899	42	170	284	201
Wiethorn 2021 [31]	Retrospective matched cohort study	Apixaban (*n* = 174)	62.6494 ± 9.6812	70	3 ± 1.4894	48	107	245	147
		Dabigatran (*n* = 59)							
		Rivaroxaban (*n* = 85)							
Coates 2021 [29]	Retrospective study	Dabigatran (*n* = 7770	62.1 ± 9.9	209	NR	NR	137	524	292
Fudim 2018 [17]	Randomized trial	Apixaban (*n* = 1035)	72.1555 ± 30.4362	164	1.95 ± 0.93	NR	NR	NR	NR
Martin 2020 [23]	Prospective observational study	apixaban and rivaroxaban (*n* = 58)	60.6466 ± 26.6078	17	NR	NR	NR	NR	NR
Choi 2017 [16]	Retrospective	Apixaban (*n* = 181)	61.7	108	NR	NR	NR	NR	NR
Deitelzweig 2020 [24]	Retrospective Observational	Apixaban (*n* = 21,242)	71.5 ± 9.9	10,215 (48.1%)	3.9 ± 1.7	NR	8068 (38.0%)	20,022 (94.3%)	11,390 (53.6%)
		Dabigatran (*n* = 7171)	69.6 ± 10.0	3138 (43.8%)	3.7 ± 1.7	NR	2487 (34.7%)	6670 (93.0%)	3778 (52.7%)
		Rivaroxaban (*n* = 29,146)	70.0 ± 10.3	13,499 (46.3%)	3.7 ± 1.7	NR	10,246 (35.2%)	27,161 (93.2%)	15,164 (52.0%)
		Warfarin (*n* = 30,902)	72.8 ± 8.8	14,928 (48.3%)	4.3 ± 1.6	NR	14,722 (47.6%)	29,379 (95.1%)	18,984 (61.4%)

### Risk of bias assessment

**Fig. 2:**
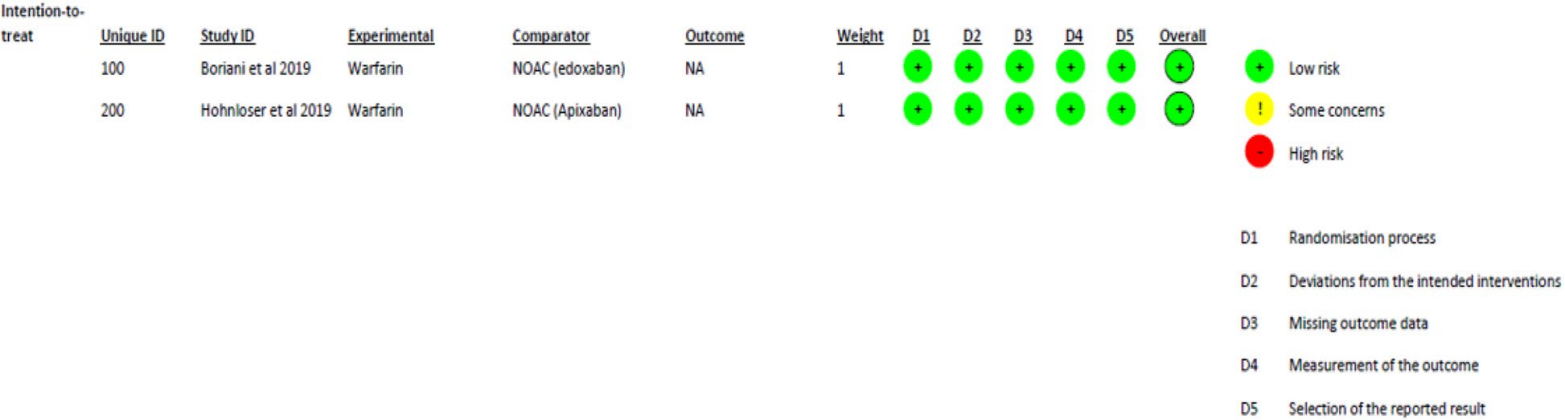
Risk of bias assessment using Risk of Bias Tool 2 (ROB-2)

Two RCTs [20, 22] were evaluated using the RoB-2 tool to assess the risk of bias [35]. The assessment led to judgments of “low risk of bias,” “some concerns,” or “high risk of bias”. Both included trials showed a low risk of bias, as shown in Fig. 2.

In the case of cohort studies, the Newcastle–Ottawa score was used for assessment, where most of the studies were of good quality and had a low risk of bias, except for two studies that were of fair quality [18, 27] and one that was of poor quality [26]. The detailed scoring system is provided in Supplementary File 2.

### Data synthesis and meta-analysis

Since our analysis included both single- and double-arm studies, we performed two different analyses for each category. First, double-arm trials have shown that patients who received NOACs had better prognosis and safety than patients who received warfarin; however, the pooled data showed heterogeneity, so we conducted a subgroup analysis to resolve the inconsistency and reduce the heterogeneity. The patients were divided according to baseline comorbidities (diabetes mellitus, hypertension, heart failure, ischaemic heart disease, peripheral artery disease, old stroke and CHA2D2-VASc score) or possible outcomes (stroke, major bleeding, and minor bleeding). Some subgroups required further sensitivity analysis, which was conducted using the “leave-one-out test”, for which all the data are provided in Supplementary 3. Finally, we performed a single-arm analysis to evaluate the outcomes of the studies with no comparison of different anticoagulation methods (only the NOAC group).

#### Double-arm studies

##### Diabetes mellitus (DM)

**Fig. 3:**
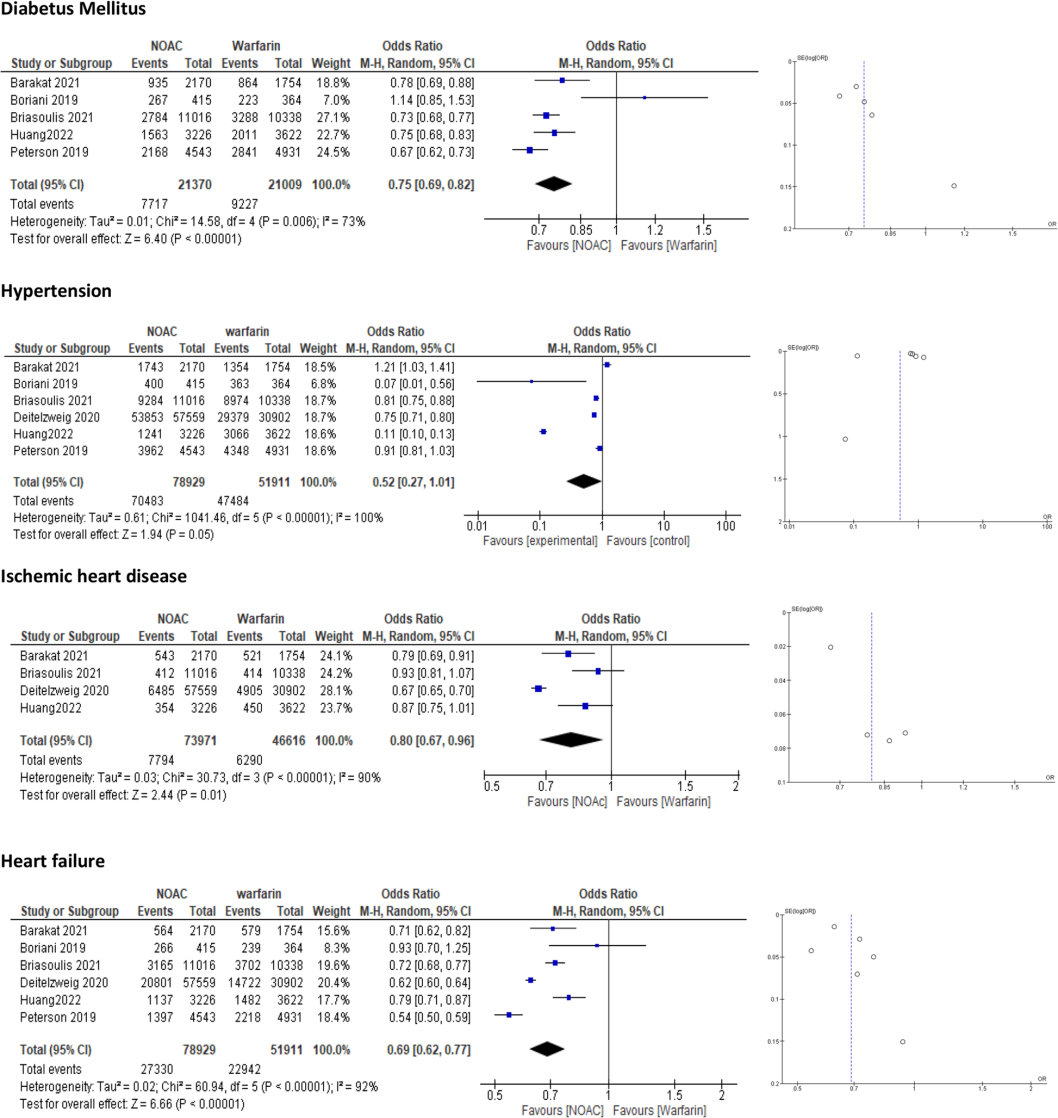
Forest plot of the random model of NOACs vs. warfarin in the subgroup of obese patients (>120 kg). NOAC = Nonvitamin K antagonist Oral Anticoagulant; CI = confidence interval

Five studies [19, 20, 25, 27, 29] included obese patients with diabetes, and the pooled data showed that this population was highly significantly more likely to benefit from NOACs than from warfarin (OR = 0.75, 95% CI = 0.69–0.82; *p* < 0.00001) (Fig. 3). However, a source of heterogeneity was observed, so we performed a sensitivity analysis with the “leave-one-out test”, which did not yield any significant difference in the overall outcome (Supplementary 3).

##### Hypertension and heart failure

Six studies [19, 20, 23, 25, 27, 29] reported hypertensive and heart failure patients in our target population. The pooled data showed a significant effect of NOACs compared with warfarin for both hypertension and heart failure (OR = 0.52, 95% CI = 0.27–1.01; *p* = 0.05 and OR = 0.69, 95% CI = 0.62–0.77]; *p* < 0.00001, respectively) (Fig. 3). The overall result in the case of hypertension was borderline, and by examination using a funnel plot, publication bias was observed.

##### Ischaemic heart disease (IHD) and peripheral artery disease (PAD)

**Fig. 4:**
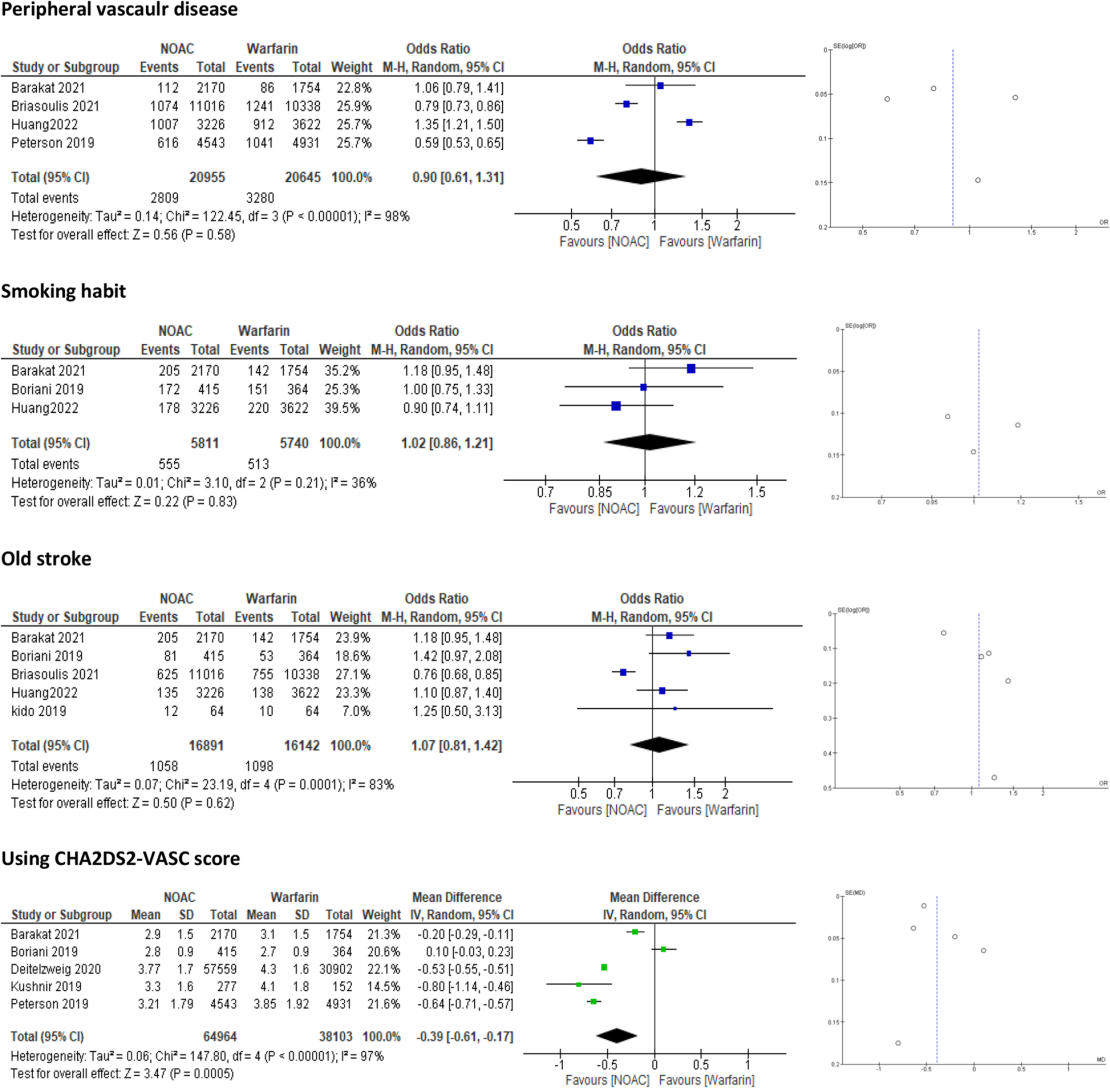
Forest plot using the random model of NOACs vs. Warfarin in the subgroup of obese patients (>120 kg). NOAC = Nonvitamin K antagonist Oral Anticoagulant; CI = confidence interval

Ischaemic heart disease (IHD) and peripheral artery disease (PAD) were defined as baseline characteristics in four studies [23, 25, 27, 29]. Pooled data showed NOACs to be superior to warfarin in overall outcome measures in the IHD group (OR = 0.80, 95% CI = 0.67–0.96]; *p* = 0.01). However, these differences were not significant in the PAD group (OR = 0.90, 95% CI = 0.61–1.31; *p* = 0.58) **(**Fig. 4**).** A source of high heterogeneity was observed in the IHD subgroup (*P* < 0.00001, I2 = 90%), so we performed a sensitivity analysis by the “leave-one-out test”, where the pooled data were homogenous, and confirmed the statistical significance of the overall outcome in favor of NOACs (Supplementary 3).

##### Smoking

Three studies [20, 27, 29] reported that smokers were not significantly different between the treatment groups (OR = 1.02, 95% CI = 0.86–1.21; *p* = 0.83) (Fig. 4).

##### Old stroke

Five studies [18, 20, 25, 27, 29] addressed the presence of old stroke in obese patients prior to treatment. The resulting data revealed a nonsignificant difference between NOACs and warfarin (OR = 1.07, 95% CI = 0.81–1.42; *p* = 0.62) **(**Fig. 4**).** However, heterogeneity was significant, so we performed the “leave-one-out test” excluding the Briasoulis et al. study [25], which caused the data to be homogenous and, interestingly, changed the result to statistical significance in favor of warfarin (OR = 1.19, 95% CI: [1.02–1.38]; *p* = 0.02) (Supplementary 3).

##### CHA2DS2 - VASc

Regarding the CHA2DS2-VASC score, five studies reported data according to the different scores [19–21, 23, 27]. The overall estimate demonstrated that NOACs are more favorable than warfarin for obese patients (MD = − 0.39, 95% CI: [− 0.61 – − 0.17]; *p* = 0.0005) (Fig. 4).

##### Outcome analysis

**Fig. 5:**
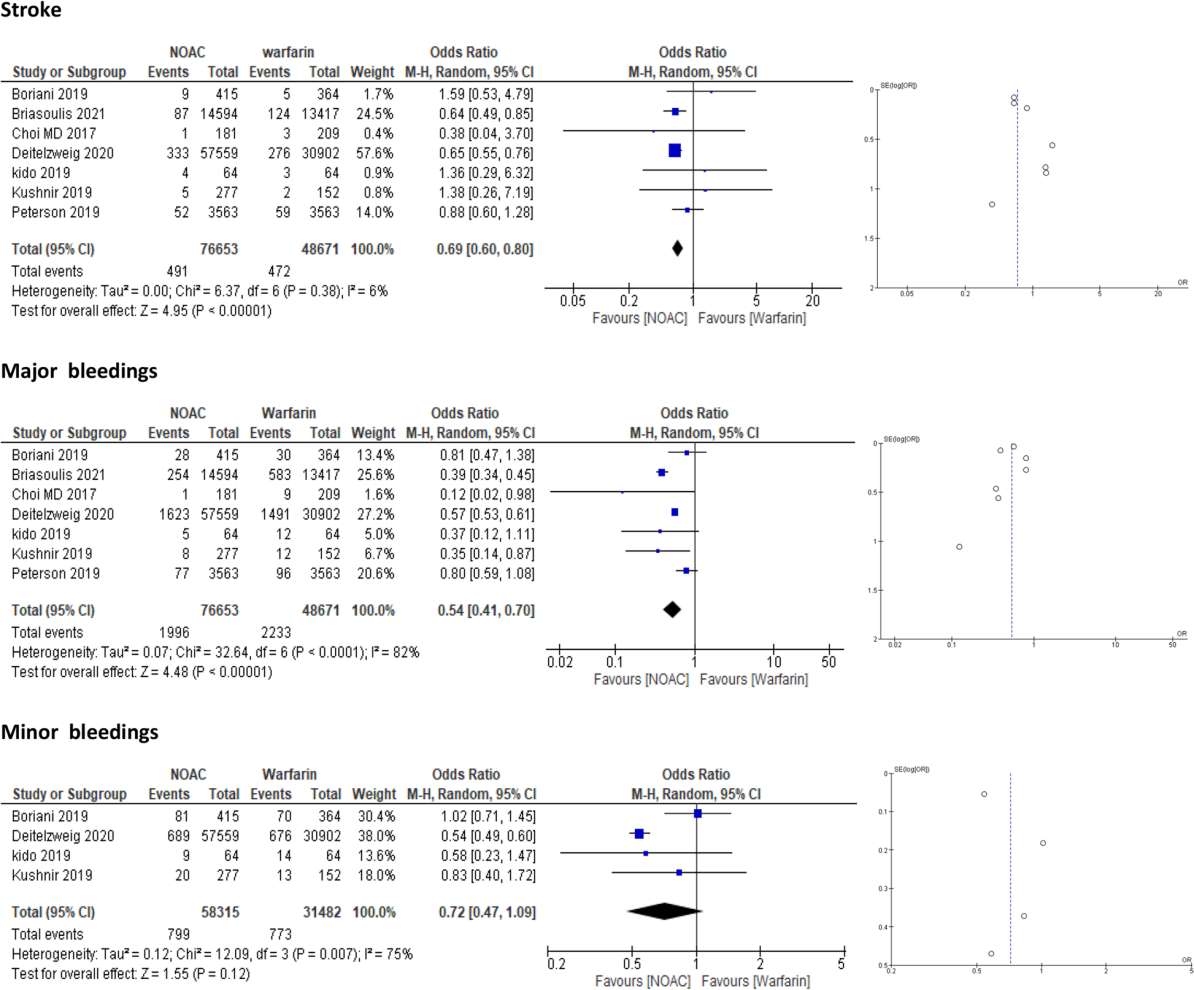
Forest plot generated using a random model of outcome in the subgroup of obese patients (> 120 kg) treated with NOACs vs. warfarin. NOAC = Nonvitamin K antagonist Oral Anticoagulant; CI = confidence interval

Seven studies [16, 18–21, 23, 25] have evaluated the outcomes of stroke, major bleeding, and minor bleeding in morbidly obese patients after anticoagulation therapy. In the case of stroke, the pooled data showed a significant difference between the NOAC group and the warfarin group (OR = 0.69, 95% CI = 0.60–0.80; *p* < 0.00001) (Fig. 5). Regarding major bleeding events, pooled data showed that patients taking NOACs had a significantly lower risk than patients taking warfarin did (OR = 0.54, 95% CI = 0.41–0.70; *p* < 0.00001) (Fig. 5). However, heterogeneity was observed, so we performed the “leave one out” test, after which the results remained significant in the NOAC group (Supplementary 3). The analysis of minor bleeding outcomes demonstrated that NOACs had a nonsignificant effect on reducing the risk of bleeding (OR = 0.72, 95% CI = 0.47–1.09; *p* = 0.12) (Fig. 5). However, after sensitivity analysis, the results became highly significant in favor of NOACs (OR = 0.55, 95% CI = 0.49–0.61; *p* < 0.00001) (Supplementary 3 Fig. 6).

#### Single-arm studies

Several of the included studies used only NOACs and not warfarin alone; only obese patients (BMI ≥ 40) and control patients (BMI < 40) were included, so we performed a single-arm analysis using Open Meta Analyst.

The pooled prevalence of diabetes mellitus and hypertension among obese patients who received nonvitamin K antagonist oral anticoagulants derived from 7 studies including a total of 2654 patients was [0.496; 95% CI: (0.397–0.595) and 0.861; 95% CI: (0.778–0.944)], respectively, for detecting DM and HTN risk **(**Supplementary 4). Other comorbidities were also analysed, and the results showed a high risk of IHD, heart failure, old cerebral vascular accidents, and stroke (represented by the CHA2D2-VASc score) in morbidly obese (Supplementary 4 Fig. 7). Regarding outcomes, including stroke as an outcome, major and minor bleeding events, and mortality rate, all presented a high risk of development among patients who received NOACs (Supplementary 4).

### Publication bias

The funnel plot presented an asymmetric pattern for baseline hypertensive obese patients treated with NOACs; therefore, the risk of publication bias cannot be excluded.

## Discussion

We conducted a systematic review and meta-analysis of a total of 17 studies [16–32] comparing the efficacy of NOACs versus warfarin in morbidly obese patients (BMI > 40 or weight > 120 kg). Our study combined observational and experimental data to include the largest number of patients possible in this category and compare outcomes.

The health impacts of AF extend beyond stroke and are known to increase mortality, myocardial infarction, heart failure incidence, hospitalization, and other cardiovascular disease risks [38]. Generally, NOACs have equivalent or superior outcomes to warfarin in nonvalvular AF patients [39]. Due to the variable distribution of the drug in the body, the anticoagulant efficacy of NOACs, which are lipophilic drugs, may be impacted by body weight [40]. Subsequently, pharmacokinetic studies have demonstrated that in overweight patients, typical fixed-dose medication concentrations decrease with increasing volume of distribution [41]. Due to drug accumulation, this could result in undesirable drug levels, a possible increase in the risk of ischaemic events, and long-term side effects that are presently not well defined. Therefore, the current meta-analysis further stresses the importance, safety, and efficacy of NOACs in individuals with AF and a high body weight (> 120 kg).

Huang [29] and colleagues reported that NOAC use was significantly associated with a greater risk of bleeding. However, after subgroup analysis, patients with a BMI ≥45 kg/m2 had a nonsignificant reduction in the risk of composite thromboembolism, an increased risk of composite bleeding, and a reduced risk of mortality when comparing dabigatran to warfarin (HR = 1.00, 95% CI = 0.45–2.23; *p* = 1), (HR = 1.31, 95% CI = 0.83–2.08]; *p* = 0.25) and (HR = 0.63, 95% CI = 0.28–1.38]; *p* = 0.24), respectively). This highlights the importance of subgroup analysis and how overweight patients may have a wrongful estimation of the effect when overweight is combined with multiple weight groups. We accordingly ensured that our analysis was based on suitable subcategorizations for each risk factor and possible outcome to eliminate any possible misinterpretations.

Regarding overall bleeding risk, a study by Bodega et al. [26] found no statistically significant differences between different body weight groups (*p* = 0.125). Nevertheless, there was a trend toward a reduction in adverse events in relation to the increase in body weight. Additionally, all-cause deaths were not significantly different among the overweight, normal, or underweight groups (*p* = 0.829). A possible explanation for this tendency is that patients with an elevated body weight may have altered medication pharmacokinetics, which decreases bleeding but does not increase thromboembolic risk [26]. Notably, the underweight patients in this study tended to be older, have poorer creatinine clearance, and be prescribed more low-dose NOACs. These results clearly show that individuals with a smaller body distribution volume should receive less medication to lower the risk of overdose and, consequently, hemorrhagic events.

The ARISTOTLE trial was a major trial on NOACs and provided many insights and post hoc analyses. In our review, two studies discussed insights from this trial. The first is a study by Fudim et al. [17], who concluded that the efficacy and safety of NOACs, specifically apixaban, in comparison to warfarin were the same for both patients with very high body weights and those without very high body weights. The high-birthweight group results were mostly insignificant except for major or clinically relevant nonmajor bleeding events (*p* value = 0.0363). Consequently, the second analysis by Hohnloser et al. [22] showed that the effectiveness and safety of apixaban compared with warfarin were maintained across different weight groups, with markedly greater decreases in major bleeding in underweight or normal AF patients than in overweight AF patients (interaction p value = 0.016). In patients with AF across the weight spectrum, including low- and very high-weight patients, apixaban appears to be advantageous to warfarin in terms of efficacy and safety for stroke prevention (interaction p value> 0.05). These conclusions all seem to favor the use of apixaban in patients with AF irrespective of body weight. Our overall outcome analysis supported these findings, where we observed a significantly reduced risk of major bleeding in favor of NOACs, including apixaban (OR = 0.54, 95% CI = 0.41–0.70]; *p* < 0.00001).

A recent Japanese meta-analysis on the obesity paradox and how the obese population may have better health profiles than individuals with a normal BMI stated that, in comparison to those in the normal-weight group, the overweight and obese groups had decreased relative risks (RRs) of 0.87 (95% CI 0.67–1.15) and 0.82 (95% CI 0.59–1.14) for the composite end points of stroke or systemic embolism (SSE), all-cause death, and cardiovascular death, respectively, even in patients receiving oral anticoagulants [42]. These findings are comparable to our findings in which we revealed an overall significant decrease in stroke incidence after receiving NOACs in the morbidly obese group (OR = 0.69, 95% CI = 0.60–0.80; *p* < 0.00001). However, we discovered a significant risk of death, with an estimated value of 0.029 (95% CI: [0.019–0.038]; (*p* < 0.001); this finding contrasts with the findings of other studies, which could be attributed to differences in confounders and ethnicity since they included only Japanese patients, while our review was not restricted to a certain race.

### Strengths and limitations

The strengths of our meta-analysis include the fact that we included very recent studies (2017–2023). The sources of heterogeneity were well explored by using random effect models and the leave out test for sensitivity analysis, which in turn showed no significant difference in most results. To our knowledge, this meta-analysis is the first to include multiple studies, including RCTs, single-arm studies, post hoc analyses, and cohort studies, to compare the efficacy of NOACs to that of warfarin in morbidly obese patients (BMI > 40 kg/m2 and/or weight > 120 kg).

The primary drawback of our meta-analysis is the variety of study designs included, which in turn caused high heterogeneity, although we addressed this issue in our analysis. Furthermore, since the design of the post hoc analysis studies does not follow the population or randomization models of statistical inference, the results of the analyses included in our review could be misleading. Finally, we considered studies written in English only, so our findings cannot be generalized. Another important limitation is that the funnel plot for studies discussing hypertension in morbidly obese patients presented asymmetry; thus, publication bias cannot be completely excluded.

### Future directions

Real-world reports on the outcomes of NOACs versus warfarin in patients with AF with a BMI > 40 kg/m2 are mostly limited by small sample sizes. Thus, for a more thorough evaluation and validation of the use of NOACs in morbid obesity, larger retrospective studies, as well as randomized, controlled prospective studies, are needed.

## Conclusion

Overall, our meta-analysis showed a generally favorable efficacy and safety profile of NOACs vs warfarin in morbidly obese patient groups (> 120 kg). Some outcomes were not statistically significant; thus, to verify these findings and compare efficacy with other treatment modalities, larger multicenter randomized controlled trials are advised to better assess their safety and efficacy in this particular weight group.

### Supplementary Information

**Additional file 1.** Search Strategy.

**Additional file 2.** New Castle Ottawa scale assessment (NOS).

**Additional file 3.** Forest plot showing the sensitivity analysis using the “leave-one-out test” in obese patients (120 kg) with diabetes mellitus. CI = confidence interval.

**Additional file 4.** Forest plot showing the prevalence of comorbid conditions in single-arm studies comparing NOACs to warfarin in morbidly obese patients.

## Data Availability

Not applicable.

## Abbreviations

NOACs: Nonvitamin K antagonist oral anticoagulants
BMI: Body mass index
AF: Atrial fibrillation
