# Supplementary material for: The safety and efficacy of nonvitamin K antagonist oral anticoagulants in morbidly obese patients with atrial fibrillation: a meta-analysis
Source: BMC Cardiovasc Disord. 2024 Jan 26;24:74. doi: 10.1186/s12872-024-03731-3 (PMC10811832; doi:10.1186/s12872-024-03731-3)
Supplement: Supplementary file 1 — Additional file 1. Search Strategy. [file 12872_2024_3731_MOESM1_ESM.docx]

**General Search Strategy**

The search terms were [“apixaban” or “Eliquis” or “dabigatran” or “Pradaxa” or “rivaroxaban” or “Xarelto” or “edoxaban” or “Savaysa” or “betrixaban” or “Bevyxxa” or “new oral anticoagulants” or “direct oral anticoagulants” or “Novel oral anticoagulants” or “New oral anticoagulants” or “DOACs” or “DOAC” or “NOACs” or “NOAC” or “non-vitamin K oral anticoagulants” or “direct factor Xa inhibitor” or “direct thrombin inhibitor” or “Nonwarfarin”] AND [“Atrial Fibrillation”] AND [“morbid obese” or “morbid obesity” or “extreme body weight” or “high body weight” or “high body mass index”]

**Cochrane Library**

Date Run: 30/09/2022 13:52:59

ID Search Hits

#1 (Direct) AND (oral) AND (anticoagulant*) 1097

#2 (Novel) AND (oral) AND (anticoagulant*) 329

#3 (New) AND (oral) AND (anticoagulant*) 925

#4 (NOAC) OR (NOACs) OR ( DOAC) OR (DOACs) 830

#5 Nonwarfarin 19

#6 (dabigatran) OR (Pradaxa) 1194

#7 (rivaroxaban) OR (Xarelto) 2158

#8 (apixaban) OR (Eliquis) 1181

#9 (edoxaban) OR (Savaysa) 712

#10 (betrixaban) OR (Bevyxxa) 126

#11 (direct factor Xa inhibitor*) 504

#12 (direct thrombin inhibitor*) 593

#13 #1 OR #2 Or #3 OR #4 OR #5 OR #6 OR #7 OR #8 OR #9 OR #10 OR #11 OR #12 5618

#14 (atrial) AND (fibrillation*) 14876

#15 #13 AND #14 2101

#16 (obese) OR (Obesity) 53448

#17 (extreme) AND ("body weight") 357

#18 (high) AND ("body weight") 13638

#19 (high) AND ("body mass index") 12349

#20 (morbid) AND (Obes*) 2758

#21 #16 OR #17 OR #18 OR #19 OR #20 68909

#22 #15 AND #21 79

**Web of Science Search Strategy (v0.1)**

# Database: Web of Science Core Collection

# Entitlements:

- WOS.SSCI: 1900 to 2022

- WOS.AHCI: 1975 to 2022

- WOS.BHCI: 2005 to 2022

- WOS.ISTP: 1990 to 2022

- WOS.ESCI: 2005 to 2022

- WOS.SCI: 1900 to 2022

- WOS.BSCI: 2005 to 2022

- WOS.ISSHP: 1990 to 2022

# Searches:

1: ((ALL=(Direct)) AND ALL=(oral)) AND ALL=(anticoagulant*) Date run: Thu Sep 29 2022 21:33:42 GMT+0200 (Eastern European Standard Time) Results: 9143

2: ((ALL=(Novel)) AND ALL=(oral)) AND ALL=(anticoagulant*) Date run: Thu Sep 29 2022 21:34:14 GMT+0200 (Eastern European Standard Time) Results: 2686

3: ((ALL=(New)) AND ALL=(oral)) AND ALL=(anticoagulant*) Date run: Thu Sep 29 2022 21:34:55 GMT+0200 (Eastern European Standard Time) Results: 6908

4: ALL=(nonwarfarin*) Date run: Thu Sep 29 2022 21:35:23 GMT+0200 (Eastern European Standard Time) Results: 25

5: ALL=(Direct factor Xa inhibitor*) Date run: Thu Sep 29 2022 21:35:51 GMT+0200 (Eastern European Standard Time) Results: 3195

6: (((ALL=(NOAC*)) OR ALL=(DOAC*)) OR ALL=(NOACs*)) OR ALL=(DOACs*) Date run: Thu Sep 29 2022 21:36:34 GMT+0200 (Eastern European Standard Time) Results: 13947

7: (ALL=(dabigatran)) OR ALL=(Pradaxa) Date run: Thu Sep 29 2022 21:37:12 GMT+0200 (Eastern European Standard Time) Results: 9971

8: (ALL=(rivaroxaban)) OR ALL=(xarelto) Date run: Thu Sep 29 2022 21:37:35 GMT+0200 (Eastern European Standard Time) Results: 10549

9: (ALL=(apixaban)) OR ALL=(eliquis) Date run: Thu Sep 29 2022 21:38:02 GMT+0200 (Eastern European Standard Time) Results: 6562

10: (ALL=(edoxaban)) OR ALL=(savaysa) Date run: Thu Sep 29 2022 21:38:21 GMT+0200 (Eastern European Standard Time) Results: 2431

11: (ALL=(betrixaban)) OR ALL=(bevyxxa) Date run: Thu Sep 29 2022 21:38:42 GMT+0200 (Eastern European Standard Time) Results: 247

12: #1 OR #2 OR #3 OR #4 OR #5 OR #6 OR #7 OR #8 OR #9 OR #10 OR #11 Date run: Thu Sep 29 2022 21:39:16 GMT+0200 (Eastern European Standard Time) Results: 34989

13: ALL=(atrial fibrillation*) Date run: Thu Sep 29 2022 21:40:25 GMT+0200 (Eastern European Standard Time) Results: 123275

14: #12 AND #13 Date run: Thu Sep 29 2022 21:40:43 GMT+0200 (Eastern European Standard Time) Results: 12316

15: ALL=(morbid obese*) Date run: Thu Sep 29 2022 21:41:36 GMT+0200 (Eastern European Standard Time) Results: 7566

16: ALL=(weight >120) Date run: Thu Sep 29 2022 21:42:18 GMT+0200 (Eastern European Standard Time) Results: 30643

17: ALL=(extreme Obese*) Date run: Thu Sep 29 2022 21:43:07 GMT+0200 (Eastern European Standard Time) Results: 1498

18: ALL=(high body mass index) Date run: Thu Sep 29 2022 21:44:59 GMT+0200 (Eastern European Standard Time) Results: 148073

19: (ALL=(obese)) OR ALL=(obesity) Date run: Thu Sep 29 2022 21:46:27 GMT+0200 (Eastern European Standard Time) Results: 553858

20: #19 OR #18 OR #15 OR #16 OR #17 Date run: Thu Sep 29 2022 21:47:45 GMT+0200 (Eastern European Standard Time) Results: 659071

21: #14 AND #20 Date run: Thu Sep 29 2022 21:48:00 GMT+0200 (Eastern European Standard Time) Results: 247

22: #14 AND #20 and Letter or Review Article or Editorial Material (Exclude – Document Types) Date run: Thu Sep 29 2022 21:49:54 GMT+0200 (Eastern European Standard Time) Results: 186

**Scopus**

Obese, AF, and Anticoagulation

( ( ( TITLE-ABS-KEY ( dabigatran OR pradaxa OR rivaroxaban OR xarelto OR apixaban OR eliquis OR edoxaban OR savaysa OR betrixaban OR bevyxxa ) OR TITLE-ABS-KEY ( direct AND oral AND anticoagulant* ) OR TITLE-ABS-KEY ( novel AND oral AND anticoagulant* ) OR TITLE-ABS-KEY ( new AND oral AND anticoagulant* ) OR TITLE-ABS-KEY ( noac OR noacs OR doac OR doacs ) OR TITLE-ABS-KEY ( nonwarfarin ) OR TITLE-ABS-KEY ( direct AND thrombin AND inhibitor* ) OR TITLE-ABS-KEY ( direct AND factor AND xa AND inhibitor* ) ) ) AND ( TITLE-ABS-KEY ( atrial AND fibrillation* ) ) ) AND ( ( TITLE-ABS-KEY ( obese ) OR TITLE-ABS-KEY ( obesity ) OR TITLE-ABS-KEY ( extreme AND "body weight" ) OR TITLE-ABS-KEY ( high AND "body weight" ) OR TITLE-ABS-KEY ( high AND "body mass index" ) OR TITLE-ABS-KEY ( morbid AND obes* ) ) )

Limited to English and article

( ( ( TITLE-ABS-KEY ( dabigatran OR pradaxa OR rivaroxaban OR xarelto OR apixaban OR eliquis OR edoxaban OR savaysa OR betrixaban OR bevyxxa ) OR TITLE-ABS-KEY ( direct AND oral AND anticoagulant* ) OR TITLE-ABS-KEY ( novel AND oral AND anticoagulant* ) OR TITLE-ABS-KEY ( new AND oral AND anticoagulant* ) OR TITLE-ABS-KEY ( noac OR noacs OR doac OR doacs ) OR TITLE-ABS-KEY ( nonwarfarin ) OR TITLE-ABS-KEY ( direct AND thrombin AND inhibitor* ) OR TITLE-ABS-KEY ( direct AND factor AND xa AND inhibitor* ) ) ) AND ( TITLE-ABS-KEY ( atrial AND fibrillation* ) ) ) AND ( ( TITLE-ABS-KEY ( obese ) OR TITLE-ABS-KEY ( obesity ) OR TITLE-ABS-KEY ( extreme AND "body weight" ) OR TITLE-ABS-KEY ( high AND "body weight" ) OR TITLE-ABS-KEY ( high AND "body mass index" ) OR TITLE-ABS-KEY ( morbid AND obes* ) ) ) AND ( LIMIT-TO ( DOCTYPE , "ar" ) ) AND ( LIMIT-TO ( LANGUAGE , "English" ) ) AND ( LIMIT-TO ( SRCTYPE , "j" ) )

PubMed

| Search number | Query | Sort By | Filters | Search Details | Results | Time |
| --- | --- | --- | --- | --- | --- | --- |
| 1 | (Direct) AND (oral) AND (anticoagulant*) | | | ("direct"[All Fields] OR "directed"[All Fields] OR "directing"[All Fields] OR "direction"[All Fields] OR "directional"[All Fields] OR "directions"[All Fields] OR "directivities"[All Fields] OR "directivity"[All Fields] OR "directs"[All Fields]) AND ("mouth"[MeSH Terms] OR "mouth"[All Fields] OR "oral"[All Fields]) AND "anticoagulant*"[All Fields] | 8,361 | 8:36:06 |
| 2 | (Novel) AND (oral) AND (anticoagulant*) | | | ("novel"[All Fields] OR "novel s"[All Fields] OR "novels"[All Fields]) AND ("mouth"[MeSH Terms] OR "mouth"[All Fields] OR "oral"[All Fields]) AND "anticoagulant*"[All Fields] | 2,754 | 8:37:08 |
| 3 | (New) AND (oral) AND (anticoagulant*) | | | "New"[All Fields] AND ("mouth"[MeSH Terms] OR "mouth"[All Fields] OR "oral"[All Fields]) AND "anticoagulant*"[All Fields] | 6,969 | 8:37:44 |
| 4 | (NOAC) OR (NOACs) OR ( DOAC) OR (DOACs) | | | "n 4 oleylcytosine arabinoside"[Supplementary Concept] OR "n 4 oleylcytosine arabinoside"[All Fields] OR "noac"[All Fields] OR "noac s"[All Fields] OR "noacs"[All Fields] OR "DOAC"[All Fields] OR "DOACs"[All Fields] | 6,510 | 8:37:58 |
| 5 | Nonwarfarin | |  | "Nonwarfarin"[All Fields] | 78 | 8:38:13 |
| 6 | (dabigatran) OR (Pradaxa) | | | "dabigatran"[MeSH Terms] OR "dabigatran"[All Fields] OR "dabigatran s"[All Fields] OR ("dabigatran"[MeSH Terms] OR "dabigatran"[All Fields] OR "pradaxa"[All Fields] OR ("dabigatran"[All Fields] AND "etexilate"[All Fields]) OR "dabigatran etexilate"[All Fields]) | 6,411 | 8:38:37 |
| 7 | (rivaroxaban) OR (Xarelto) | | | "rivaroxaban"[MeSH Terms] OR "rivaroxaban"[All Fields] OR "rivaroxaban"[MeSH Terms] OR "rivaroxaban"[All Fields] OR "xarelto"[All Fields] | 7,639 | 8:38:55 |
| 8 | (apixaban) OR (Eliquis) | | | "apixaban"[Supplementary Concept] OR "apixaban"[All Fields] OR "apixaban s"[All Fields] OR "apixaban"[Supplementary Concept] OR "apixaban"[All Fields] OR "eliquis"[All Fields] OR "apixaban s"[All Fields] | 5,077 | 8:39:09 |
| 9 | (edoxaban) OR (Savaysa) | | | "edoxaban"[Supplementary Concept] OR "edoxaban"[All Fields] OR "edoxaban"[Supplementary Concept] OR "edoxaban"[All Fields] OR "savaysa"[All Fields] | 2,058 | 8:39:29 |
| 10 | (betrixaban) OR (Bevyxxa) | | | "betrixaban"[Supplementary Concept] OR "betrixaban"[All Fields] OR "betrixaban"[Supplementary Concept] OR "betrixaban"[All Fields] OR "bevyxxa"[All Fields] | 217 | 8:40:06 |
| 11 | (direct factor Xa inhibitor*) | | | ("direct"[All Fields] OR "directed"[All Fields] OR "directing"[All Fields] OR "direction"[All Fields] OR "directional"[All Fields] OR "directions"[All Fields] OR "directivities"[All Fields] OR "directivity"[All Fields] OR "directs"[All Fields]) AND ("factor xa"[MeSH Terms] OR ("factor"[All Fields] AND "xa"[All Fields]) OR "factor xa"[All Fields]) AND "inhibitor*"[All Fields] | 3,189 | 8:40:39 |
| 12 | (direct thrombin inhibitor*) | | | ("direct"[All Fields] OR "directed"[All Fields] OR "directing"[All Fields] OR "direction"[All Fields] OR "directional"[All Fields] OR "directions"[All Fields] OR "directivities"[All Fields] OR "directivity"[All Fields] OR "directs"[All Fields]) AND ("thrombin"[MeSH Terms] OR "thrombin"[All Fields] OR "thrombin s"[All Fields] OR "thrombine"[All Fields] OR "thrombins"[All Fields]) AND "inhibitor*"[All Fields] | 4,672 | 8:41:05 |
| 14 | #1 OR #2 OR #3 OR #4 OR #5 OR #6 OR #7 OR #8 OR #9 OR #10 OR #11 OR #12 | | | (("direct"[All Fields] OR "directed"[All Fields] OR "directing"[All Fields] OR "direction"[All Fields] OR "directional"[All Fields] OR "directions"[All Fields] OR "directivities"[All Fields] OR "directivity"[All Fields] OR "directs"[All Fields]) AND ("mouth"[MeSH Terms] OR "mouth"[All Fields] OR "oral"[All Fields]) AND "anticoagulant*"[All Fields]) OR (("novel"[All Fields] OR "novel s"[All Fields] OR "novels"[All Fields]) AND ("mouth"[MeSH Terms] OR "mouth"[All Fields] OR "oral"[All Fields]) AND "anticoagulant*"[All Fields]) OR ("New"[All Fields] AND ("mouth"[MeSH Terms] OR "mouth"[All Fields] OR "oral"[All Fields]) AND "anticoagulant*"[All Fields]) OR ("n 4 oleylcytosine arabinoside"[Supplementary Concept] OR "n 4 oleylcytosine arabinoside"[All Fields] OR "noac"[All Fields] OR ("noac s"[All Fields] OR "noacs"[All Fields]) OR "DOAC"[All Fields] OR "DOACs"[All Fields]) OR "Nonwarfarin"[All Fields] OR ("dabigatran"[MeSH Terms] OR "dabigatran"[All Fields] OR "dabigatran s"[All Fields] OR ("dabigatran"[MeSH Terms] OR "dabigatran"[All Fields] OR "pradaxa"[All Fields] OR ("dabigatran"[All Fields] AND "etexilate"[All Fields]) OR "dabigatran etexilate"[All Fields])) OR ("rivaroxaban"[MeSH Terms] OR "rivaroxaban"[All Fields] OR ("rivaroxaban"[MeSH Terms] OR "rivaroxaban"[All Fields] OR "xarelto"[All Fields])) OR ("apixaban"[Supplementary Concept] OR "apixaban"[All Fields] OR "apixaban s"[All Fields] OR ("apixaban"[Supplementary Concept] OR "apixaban"[All Fields] OR "eliquis"[All Fields] OR "apixaban s"[All Fields])) OR ("edoxaban"[Supplementary Concept] OR "edoxaban"[All Fields] OR ("edoxaban"[Supplementary Concept] OR "edoxaban"[All Fields] OR "savaysa"[All Fields])) OR ("betrixaban"[Supplementary Concept] OR "betrixaban"[All Fields] OR ("betrixaban"[Supplementary Concept] OR "betrixaban"[All Fields] OR "bevyxxa"[All Fields])) OR (("direct"[All Fields] OR "directed"[All Fields] OR "directing"[All Fields] OR "direction"[All Fields] OR "directional"[All Fields] OR "directions"[All Fields] OR "directivities"[All Fields] OR "directivity"[All Fields] OR "directs"[All Fields]) AND ("factor xa"[MeSH Terms] OR ("factor"[All Fields] AND "xa"[All Fields]) OR "factor xa"[All Fields]) AND "inhibitor*"[All Fields]) OR (("direct"[All Fields] OR "directed"[All Fields] OR "directing"[All Fields] OR "direction"[All Fields] OR "directional"[All Fields] OR "directions"[All Fields] OR "directivities"[All Fields] OR "directivity"[All Fields] OR "directs"[All Fields]) AND ("thrombin"[MeSH Terms] OR "thrombin"[All Fields] OR "thrombin s"[All Fields] OR "thrombine"[All Fields] OR "thrombins"[All Fields]) AND "inhibitor*"[All Fields]) | 25,935 | 8:42:03 |
| 15 | (atrial) AND (fibrillation*) | | | ("atrialisation"[All Fields] OR "atrialization"[All Fields] OR "atrialized"[All Fields] OR "atrially"[All Fields] OR "heart atria"[MeSH Terms] OR ("heart"[All Fields] AND "atria"[All Fields]) OR "heart atria"[All Fields] OR "atrial"[All Fields]) AND "fibrillation*"[All Fields] | 100,990 | 8:42:43 |
| 16 | (#14) AND (#15) | |  | ((("direct"[All Fields] OR "directed"[All Fields] OR "directing"[All Fields] OR "direction"[All Fields] OR "directional"[All Fields] OR "directions"[All Fields] OR "directivities"[All Fields] OR "directivity"[All Fields] OR "directs"[All Fields]) AND ("mouth"[MeSH Terms] OR "mouth"[All Fields] OR "oral"[All Fields]) AND "anticoagulant*"[All Fields]) OR (("novel"[All Fields] OR "novel s"[All Fields] OR "novels"[All Fields]) AND ("mouth"[MeSH Terms] OR "mouth"[All Fields] OR "oral"[All Fields]) AND "anticoagulant*"[All Fields]) OR ("New"[All Fields] AND ("mouth"[MeSH Terms] OR "mouth"[All Fields] OR "oral"[All Fields]) AND "anticoagulant*"[All Fields]) OR ("n 4 oleylcytosine arabinoside"[Supplementary Concept] OR "n 4 oleylcytosine arabinoside"[All Fields] OR "noac"[All Fields] OR ("noac s"[All Fields] OR "noacs"[All Fields]) OR "DOAC"[All Fields] OR "DOACs"[All Fields]) OR "Nonwarfarin"[All Fields] OR ("dabigatran"[MeSH Terms] OR "dabigatran"[All Fields] OR "dabigatran s"[All Fields] OR ("dabigatran"[MeSH Terms] OR "dabigatran"[All Fields] OR "pradaxa"[All Fields] OR ("dabigatran"[All Fields] AND "etexilate"[All Fields]) OR "dabigatran etexilate"[All Fields])) OR ("rivaroxaban"[MeSH Terms] OR "rivaroxaban"[All Fields] OR ("rivaroxaban"[MeSH Terms] OR "rivaroxaban"[All Fields] OR "xarelto"[All Fields])) OR ("apixaban"[Supplementary Concept] OR "apixaban"[All Fields] OR "apixaban s"[All Fields] OR ("apixaban"[Supplementary Concept] OR "apixaban"[All Fields] OR "eliquis"[All Fields] OR "apixaban s"[All Fields])) OR ("edoxaban"[Supplementary Concept] OR "edoxaban"[All Fields] OR ("edoxaban"[Supplementary Concept] OR "edoxaban"[All Fields] OR "savaysa"[All Fields])) OR ("betrixaban"[Supplementary Concept] OR "betrixaban"[All Fields] OR ("betrixaban"[Supplementary Concept] OR "betrixaban"[All Fields] OR "bevyxxa"[All Fields])) OR (("direct"[All Fields] OR "directed"[All Fields] OR "directing"[All Fields] OR "direction"[All Fields] OR "directional"[All Fields] OR "directions"[All Fields] OR "directivities"[All Fields] OR "directivity"[All Fields] OR "directs"[All Fields]) AND ("factor xa"[MeSH Terms] OR ("factor"[All Fields] AND "xa"[All Fields]) OR "factor xa"[All Fields]) AND "inhibitor*"[All Fields]) OR (("direct"[All Fields] OR "directed"[All Fields] OR "directing"[All Fields] OR "direction"[All Fields] OR "directional"[All Fields] OR "directions"[All Fields] OR "directivities"[All Fields] OR "directivity"[All Fields] OR "directs"[All Fields]) AND ("thrombin"[MeSH Terms] OR "thrombin"[All Fields] OR "thrombin s"[All Fields] OR "thrombine"[All Fields] OR "thrombins"[All Fields]) AND "inhibitor*"[All Fields])) AND (("atrialisation"[All Fields] OR "atrialization"[All Fields] OR "atrialized"[All Fields] OR "atrially"[All Fields] OR "heart atria"[MeSH Terms] OR ("heart"[All Fields] AND "atria"[All Fields]) OR "heart atria"[All Fields] OR "atrial"[All Fields]) AND "fibrillation*"[All Fields]) | 9,345 | 9:21:21 |
| 17 | (obese) OR (Obesity) | | | "obeses"[All Fields] OR "obesity"[MeSH Terms] OR "obesity"[All Fields] OR "obese"[All Fields] OR "obesities"[All Fields] OR "obesity s"[All Fields] OR "obeses"[All Fields] OR "obesity"[MeSH Terms] OR "obesity"[All Fields] OR "obese"[All Fields] OR "obesities"[All Fields] OR "obesity s"[All Fields] | 431,532 | 9:22:09 |
| 18 | (extreme) AND ("body weight") | | | ("extreme"[All Fields] OR "extremely"[All Fields] OR "extremes"[All Fields]) AND "body weight"[All Fields] | 3,929 | 9:22:33 |
| 19 | (high) AND ("body weight") | | | "high"[All Fields] AND "body weight"[All Fields] | 78,144 | 9:22:51 |
| 20 | (high) AND ("body mass index") | | | "high"[All Fields] AND "body mass index"[All Fields] | 77,617 | 9:23:08 |
| 21 | (morbid) AND (Obes*) | | | ("epidemiology"[MeSH Subheading] OR "epidemiology"[All Fields] OR "morbidity"[All Fields] OR "morbidity"[MeSH Terms] OR "morbid"[All Fields] OR "morbidities"[All Fields] OR "morbids"[All Fields]) AND "obes*"[All Fields] | 155,458 | 9:23:26 |
| 22 | ((((#17) OR (#18)) OR (#19)) OR (#20)) OR (#21) | | | "obeses"[All Fields] OR "obesity"[MeSH Terms] OR "obesity"[All Fields] OR "obese"[All Fields] OR "obesities"[All Fields] OR "obesity s"[All Fields] OR ("obeses"[All Fields] OR "obesity"[MeSH Terms] OR "obesity"[All Fields] OR "obese"[All Fields] OR "obesities"[All Fields] OR "obesity s"[All Fields]) OR (("extreme"[All Fields] OR "extremely"[All Fields] OR "extremes"[All Fields]) AND "body weight"[All Fields]) OR ("high"[All Fields] AND "body weight"[All Fields]) OR ("high"[All Fields] AND "body mass index"[All Fields]) OR (("epidemiology"[MeSH Subheading] OR "epidemiology"[All Fields] OR "morbidity"[All Fields] OR "morbidity"[MeSH Terms] OR "morbid"[All Fields] OR "morbidities"[All Fields] OR "morbids"[All Fields]) AND "obes*"[All Fields]) | 537,400 | 9:24:16 |
| 23 | (#16) AND (#22) | |  | ((("direct"[All Fields] OR "directed"[All Fields] OR "directing"[All Fields] OR "direction"[All Fields] OR "directional"[All Fields] OR "directions"[All Fields] OR "directivities"[All Fields] OR "directivity"[All Fields] OR "directs"[All Fields]) AND ("mouth"[MeSH Terms] OR "mouth"[All Fields] OR "oral"[All Fields]) AND "anticoagulant*"[All Fields]) OR (("novel"[All Fields] OR "novel s"[All Fields] OR "novels"[All Fields]) AND ("mouth"[MeSH Terms] OR "mouth"[All Fields] OR "oral"[All Fields]) AND "anticoagulant*"[All Fields]) OR ("New"[All Fields] AND ("mouth"[MeSH Terms] OR "mouth"[All Fields] OR "oral"[All Fields]) AND "anticoagulant*"[All Fields]) OR ("n 4 oleylcytosine arabinoside"[Supplementary Concept] OR "n 4 oleylcytosine arabinoside"[All Fields] OR "noac"[All Fields] OR ("noac s"[All Fields] OR "noacs"[All Fields]) OR "DOAC"[All Fields] OR "DOACs"[All Fields]) OR "Nonwarfarin"[All Fields] OR ("dabigatran"[MeSH Terms] OR "dabigatran"[All Fields] OR "dabigatran s"[All Fields] OR ("dabigatran"[MeSH Terms] OR "dabigatran"[All Fields] OR "pradaxa"[All Fields] OR ("dabigatran"[All Fields] AND "etexilate"[All Fields]) OR "dabigatran etexilate"[All Fields])) OR ("rivaroxaban"[MeSH Terms] OR "rivaroxaban"[All Fields] OR ("rivaroxaban"[MeSH Terms] OR "rivaroxaban"[All Fields] OR "xarelto"[All Fields])) OR ("apixaban"[Supplementary Concept] OR "apixaban"[All Fields] OR "apixaban s"[All Fields] OR ("apixaban"[Supplementary Concept] OR "apixaban"[All Fields] OR "eliquis"[All Fields] OR "apixaban s"[All Fields])) OR ("edoxaban"[Supplementary Concept] OR "edoxaban"[All Fields] OR ("edoxaban"[Supplementary Concept] OR "edoxaban"[All Fields] OR "savaysa"[All Fields])) OR ("betrixaban"[Supplementary Concept] OR "betrixaban"[All Fields] OR ("betrixaban"[Supplementary Concept] OR "betrixaban"[All Fields] OR "bevyxxa"[All Fields])) OR (("direct"[All Fields] OR "directed"[All Fields] OR "directing"[All Fields] OR "direction"[All Fields] OR "directional"[All Fields] OR "directions"[All Fields] OR "directivities"[All Fields] OR "directivity"[All Fields] OR "directs"[All Fields]) AND ("factor xa"[MeSH Terms] OR ("factor"[All Fields] AND "xa"[All Fields]) OR "factor xa"[All Fields]) AND "inhibitor*"[All Fields]) OR (("direct"[All Fields] OR "directed"[All Fields] OR "directing"[All Fields] OR "direction"[All Fields] OR "directional"[All Fields] OR "directions"[All Fields] OR "directivities"[All Fields] OR "directivity"[All Fields] OR "directs"[All Fields]) AND ("thrombin"[MeSH Terms] OR "thrombin"[All Fields] OR "thrombin s"[All Fields] OR "thrombine"[All Fields] OR "thrombins"[All Fields]) AND "inhibitor*"[All Fields])) AND (("atrialisation"[All Fields] OR "atrialization"[All Fields] OR "atrialized"[All Fields] OR "atrially"[All Fields] OR "heart atria"[MeSH Terms] OR ("heart"[All Fields] AND "atria"[All Fields]) OR "heart atria"[All Fields] OR "atrial"[All Fields]) AND "fibrillation*"[All Fields]) AND ("obeses"[All Fields] OR "obesity"[MeSH Terms] OR "obesity"[All Fields] OR "obese"[All Fields] OR "obesities"[All Fields] OR "obesity s"[All Fields] OR ("obeses"[All Fields] OR "obesity"[MeSH Terms] OR "obesity"[All Fields] OR "obese"[All Fields] OR "obesities"[All Fields] OR "obesity s"[All Fields]) OR (("extreme"[All Fields] OR "extremely"[All Fields] OR "extremes"[All Fields]) AND "body weight"[All Fields]) OR ("high"[All Fields] AND "body weight"[All Fields]) OR ("high"[All Fields] AND "body mass index"[All Fields]) OR (("epidemiology"[MeSH Subheading] OR "epidemiology"[All Fields] OR "morbidity"[All Fields] OR "morbidity"[MeSH Terms] OR "morbid"[All Fields] OR "morbidities"[All Fields] OR "morbids"[All Fields]) AND "obes*"[All Fields])) | 205 | 9:24:38 |
