# Supplementary material for: The safety and efficacy of nonvitamin K antagonist oral anticoagulants in morbidly obese patients with atrial fibrillation: a meta-analysis
Source: BMC Cardiovasc Disord. 2024 Jan 26;24:74. doi: 10.1186/s12872-024-03731-3 (PMC10811832; doi:10.1186/s12872-024-03731-3)
Supplement: Supplementary file 2 — Additional file 2. New Castle Ottawa scale assessment (NOS). [file 12872_2024_3731_MOESM2_ESM.docx]

Supplementary File 2 New Castle Ottawa scale assessment (NOS)

|  |  | **Selection** | | | | **Comparability** | **Outcome** | | | **Quality Score** |
| --- | --- | --- | --- | --- | --- | --- | --- | --- | --- | --- |
| **First Author** | **Year** | **Representativeness of the exposed cohort** | **Selection of the non exposed cohort** | **Ascertainment of exposure** | **Demonstration that outcome of interest was not present at start of study** | **Comparability of cohorts on the basis of the design or analysis** | **Assessment of outcome** | **Was follow-up long enough for outcomes to occur** | **Adequacy of follow up of cohorts** |  |
| **Alexandros Briasoulis** | **2021** | ***** | ***** | ***** |  | ****** | ***** | ***** | ***** | **Good Quality** |
| **Cheng-Wei Huang** | **2021** | ***** | ***** | ***** | ***** | ****** | ***** | ***** | ***** | **Good Quality** |
| **Eric D. Peterson** | **2019** | ***** | ***** | ***** | ***** | ****** | ***** |  | ***** | **Good Quality** |
| **Bego˜na Navarro-Almenzar** | **2021** |  | ***** | ***** | ***** | ****** | ***** | ***** | ***** | **Good Quality** |
| **Amr F. Barakat** | **2021** | ***** |  | ***** | ***** | ***** | ***** | ***** | ***** | **Fair Quality** |
| **Margarita Kushnir** | **2019** | ***** | ***** | ***** | ***** | ****** | ***** | ***** | ***** | **Good Quality** |
| **Francesca Bodega1** | **2021** |  |  | ***** | ***** |  | ***** | ***** | ***** | **Poor Quality** |
| **Kazuhiko Kido** | **2018** |  |  | ***** | ***** | ***** | ***** | ***** | ***** | **Fair Quality** |
| **Khalid Al Sulaiman** | **2022** | ***** | ***** | ***** | ***** | ****** | ***** | ***** | ***** | **Good Quality** |
| **Cavan P. O’Kane** | **2021** | ***** | ***** | ***** | ***** | ****** | ***** | ***** | ***** | **Good Quality** |
| **Eryne E. Wiethorn** | **2021** | ***** | ***** | ***** | ***** | ****** | ***** | ***** | ***** | **Good Quality** |
| **Jamie Coates** | **2021** | ***** | ***** | ***** | ***** | ****** | ***** | ***** | ***** | **Good Quality** |
| **Anne Céline Martin** | **2020** | ***** |  | ***** | ***** |  | ***** | ***** | ***** | **Poor Quality** |
| **Yun Choi** | **2017** | ***** | ***** | ***** | ***** | ****** | ***** | ***** | ***** | **Good Quality** |
| **Steve Deitelzweig** | **2020** | ***** | ***** | ***** | ***** | ****** | ***** | ***** | ***** | **Good Quality** |
