# Supplementary figures and images for: The safety and efficacy of nonvitamin K antagonist oral anticoagulants in morbidly obese patients with atrial fibrillation: a meta-analysis
Source: BMC Cardiovasc Disord. 2024 Jan 26;24:74. doi: 10.1186/s12872-024-03731-3 (PMC10811832; doi:10.1186/s12872-024-03731-3)

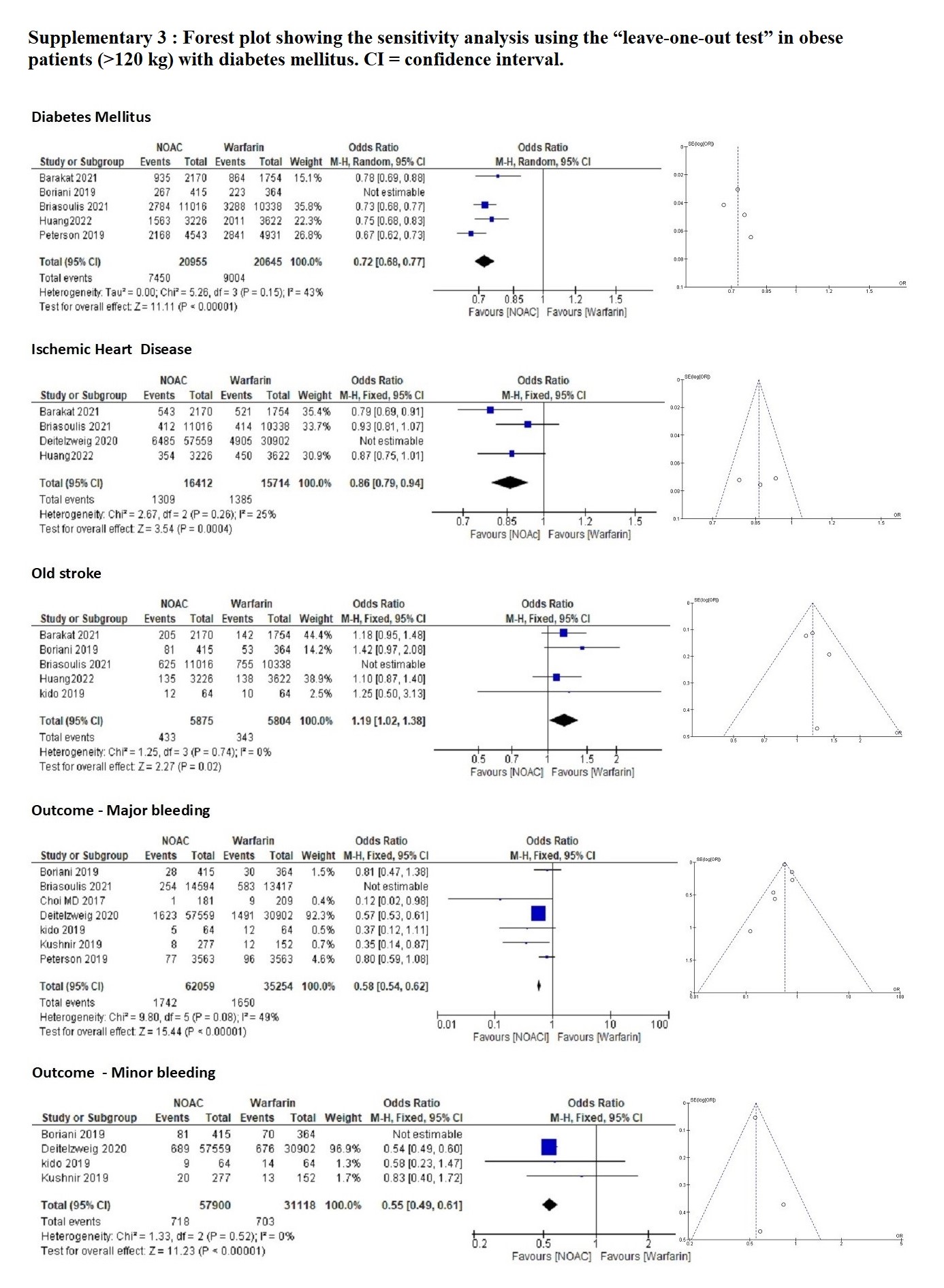

Supplement: Supplementary file 3 — Additional file 3. Forest plot showing the sensitivity analysis using the “leave-one-out test” in obese patients (120 kg) with diabetes mellitus. CI = confidence interval. [file 12872_2024_3731_MOESM3_ESM.jpg]

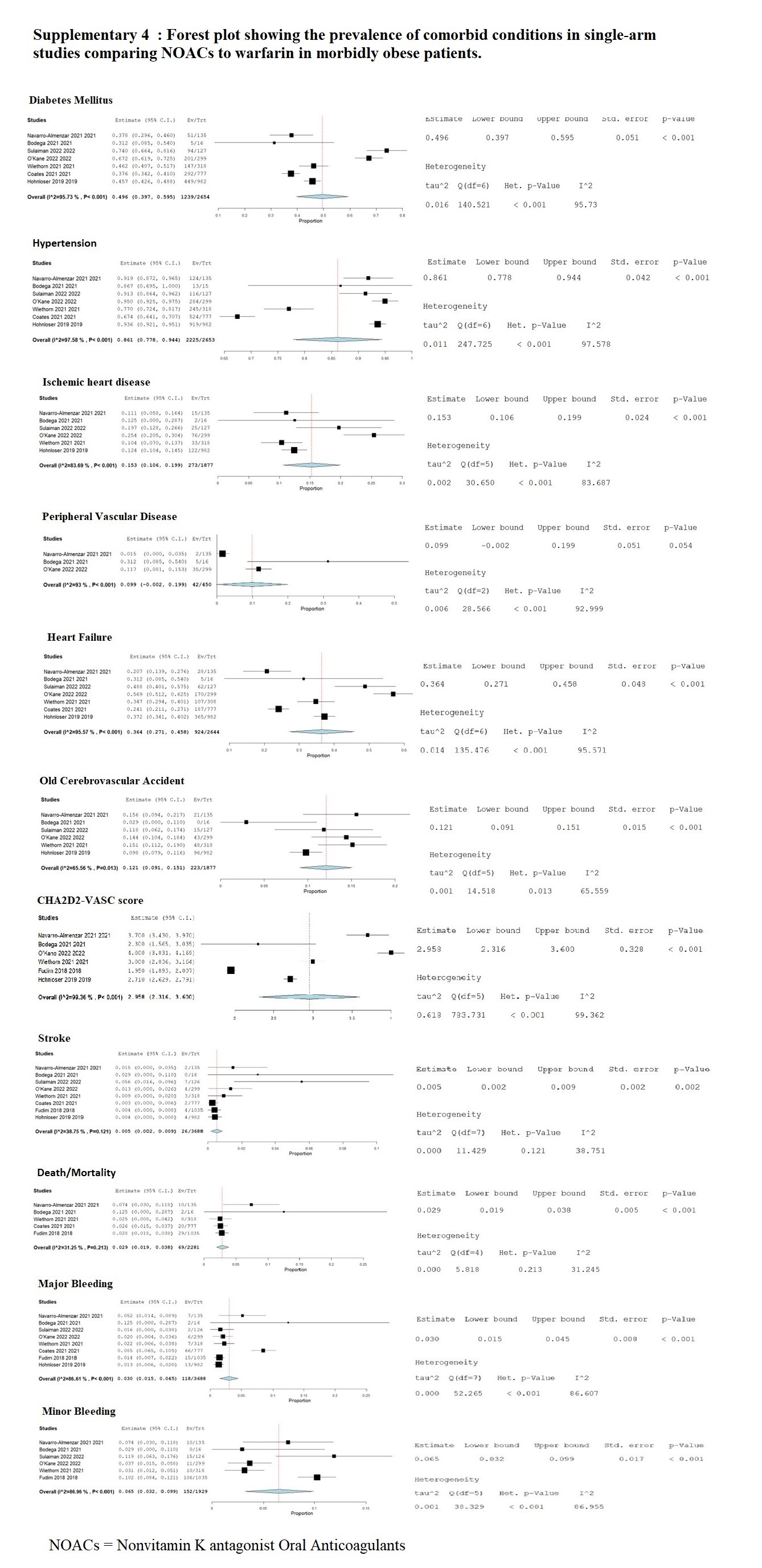

Supplement: Supplementary file 4 — Additional file 4. Forest plot showing the prevalence of comorbid conditions in single-arm studies comparing NOACs to warfarin in morbidly obese patients. [file 12872_2024_3731_MOESM4_ESM.jpg]
